# Distribution of acquired antibiotic resistance genes among *Enterococcus* spp. isolated from a hospital in Baotou, China

**DOI:** 10.1186/s13104-019-4064-z

**Published:** 2019-01-15

**Authors:** Yingjie Tian, Hui Yu, Zhanli Wang

**Affiliations:** The Second Affiliated Hospital, Baotou Medical College, 30 Hude Mulin Street, Baotou, 014030 China

**Keywords:** *Enterococcus*, Drug resistance, Antibiotic resistance gene, Infection

## Abstract

**Objective:**

This study investigated the distribution of acquired antibiotic resistance genes in *Enterococcus* species isolated from clinical patients in Baotou, China.

**Result:**

A total of 73 enterococca lisolates from clinical samples were collected from December 2016 to September 2017. Of the 73 enterococcal isolates, 36 (49.3%), 35 (47.9%), 1 (1.4%), and 1 (1.4%) were identified as *E. faecium*, *E. faecalis*, *E. gallinarum*, and *E. raffinosus*, respectively. The resistance rates of the enterococci to nitrofurantoin, tetracycline, gentamicin (high-level), ampicillin, ciprofloxacin and erythromycin were 24.7%, 49.3%, 50.7%, 54.8%, 74.0% and 89.0%, respectively. The most prevalent aminoglycoside resistance genes were *aac(6′)*-*Ie*-*aph(2″)*-*Ia* (64.9%) and *aph(3′)IIIa* (64.9%). The most common erythromycin ribosome methylation gene was *erm(B)* (67.7%), followed by e*rm(A)* (4.6%) and e*rm(C)* (1.5%). The tetracycline resistance gene *tetM* was found to be present in 100.0% of the tetracycline-resistant strains of enterococci. Thus, *E. faecium* and *E. faecalis* were identified as the species of greatest clinical importance associated with hospital-acquired enterococcal infections in Baotou, China. The antimicrobial resistance genes *aac(6′)*-*Ie*-*aph(2″)*-*Ia, aph(3′)IIIa, tetM,* and *erm(B)* were significantly more prevalent among the enterococcal isolates. Therefore, action should be taken to monitor drug resistance and antimicrobial resistance genes to manage multi-drug-resistant enterococcal infections.

**Electronic supplementary material:**

The online version of this article (10.1186/s13104-019-4064-z) contains supplementary material, which is available to authorized users.

## Introduction

Enterococci have long been considered symbiotic organisms of humans that have the potential to accidentally invade the host [1]. Recently, *Enterococcus* species have emerged as important pathogens causing hospital-acquired and community-acquired abdominal infections [2]. The common infection sites are the urinary tract, surgical sites, respiratory tract, gastrointestinal tract, skin and soft tissue. In addition, the incidence of infections caused by multi-drug-resistant isolates of enterococci is increasing worldwide, which is a serious problem for clinical anti-infective therapy [3].

Many studies have reported that enterococci are resistant to a wide range of antimicrobial agents via intrinsic and acquired mechanisms [4]. The mechanism underlying the acquisition of antibiotic resistance genes has been recognized for several decades. High-level aminoglycoside resistance is primarily due to the acquisition of genes encoding aminoglycoside-modifying enzymes (AMEs), such as *aac(6′)*-*Ie*-*aph(2″)*-*Ia, aph(2″)*-*Ib, aph(2″)*-*Ic, aph(2″)*-*Id,* and *aph(3′)IIIa* [5]. Erythromycin resistance among enterococci is associated with the presence of erythromycin resistance methylase (*erm*) genes, such as *erm(A), erm(B),* and *erm(C)*. The predominant *erm* gene in erythromycin-resistant isolates of enterococci is the *erm(B)* gene, which encodes the ribosomal RNA methylase [6]. Moreover, there are currently over 40 different acquired tetracycline resistance genes recognized. The *tet(M)* gene is the tetracycline resistance gene with the broadest host range [7]. The protein encoded by the *tetM* gene blocks the binding of tetracycline to the ribosome by combining with the 50S ribosomal subunit, causing a conformational change in the ribosome that subsequently produces drug resistance.

The difficulty in treating enterococcal infections is associated with antimicrobial resistance. Therefore, the study of the distribution of resistance genes and resistance mechanisms of enterococci to guide clinical treatment is particularly important. The aim of the present study was to determine the antibiotic susceptibility of enterococci, examine the presence of genes encoding AMEs (including *aac(6′)*-*Ie*-*aph(2″)*-*Ia*, *aph(2″)*-*Ib*, *aph(2″)*-*Ic, aph(2″)*-*Id*, and *aph(3′)*-*IIIa*) and the *erm* factors (including *erm(A), erm(B),* and *erm(C)*), and identify the *tet(M)* gene of enterococcal isolates from clinical patients in Baotou, China.

## Main text

### Methods

#### Bacterial strains and identification

A total of 73 clinical isolates of enterococci were collected from the Second Affiliated Hospital of Baotou Medical College in Baotou, China, between December 2016 and September 2017. Duplicate and contaminated isolates were excluded from the study. Institutional ethical clearance was obtained. Conventional biochemical tests and the Phoenix100 automatic system (BD, USA) were used to identify the isolates as enterococci. Identifications of *E. faecalis*, *E. faecium* and the other strains were further confirmed via PCR analysis as described previously [8].

#### Susceptibility testing

Antimicrobial susceptibility testing was performed by using the disc diffusion method according to the standards and interpretive criteria described by the Clinical and Laboratory Standards Institute (CLSI) [9]. The following drugs were tested: gentamicin (120 μg), erythromycin (15 μg), vancomycin (30 μg), teicoplanin (30 μg), ciprofloxacin (5 μg), ampicillin (10 μg), linezolid (30 μg), tetracycline (30 μg), and rifampin (5 μg). *E. faecalis* ATCC29212 was used as a reference strain.

#### Amplification of antimicrobial resistance genes

Total DNA was extracted from enterococci according to the instruction manuals of commercial DNA extraction kits (Tiangen Biotech Co., Ltd., Beijing, China). The presence of the antimicrobial resistance genes was detected by PCR (see Additional file 1: Table S1) [10–13]. PCR amplification was performed using 5 μl of template DNA, 2 μl of each primer (100 pmol), and 25 μl of 2 × Taq PCR MasterMix (Solarbio Science & Technology Co., Ltd., Beijing, China) in a total reaction volume of 50 μl. The PCR conditions consisted of an initial denaturation step at 94 °C for 3 min, followed by 35 cycles of denaturation at 94 °C for 30 s, annealing for 30 s, and elongation at 72 °C for 1 min. A final extension step was carried out at 72 °C for 5 min.

#### Statistical analysis

All statistical analyses were performed using Statistical Package for Social Sciences (SPSS) software (version 17.0). Probability values (p) of < 0.05 were considered statistically significant.

### Results

#### Identification of Enterococcus species

A total of 73 enterococcus isolates were obtained from different clinical samples as follows: urine (n = 36, 49.3%), pus (n = 11, 15.1%), bile (n = 10, 13.7%), wounds (n = 8, 11.0%), hydrothorax (n = 3, 4.1%), blood (n = 2, 2.7%), and others (n = 3, 4.1%). Among the 73 strains of enterococci, *E. faecium* (n = 36, 49.3%) and *E. faecalis* (n = 35, 47.9%) were identified as the dominant species presented in this study, along with *E. gallinarum* (n = 1, 1.4%) and *E. raffinosus* (n = 1, 1.4%).

#### Antimicrobial susceptibility

Among the 9 antibacterial agents tested, the frequencies of resistance of enterococci to nitrofurantoin, tetracycline, gentamicin (high-level), ampicillin, ciprofloxacin and erythromycin were 24.7%, 49.3%, 50.7%, 54.8%, 74.0% and 89.0%, respectively (see Additional file 2: Table S2). All strains were sensitive to teicoplanin, vancomycin and linezolid. There was a significant difference in the resistance to ampicillin between *E. faecium* and *E. faecalis* species (p < 0.05). However, no significant differences in resistance rates to tetracycline (p = 0.059), gentamicin (p = 0.479), ciprofloxacin (p = 0.173), and erythromycin (p = 0.226) were observed.

#### Distribution of aminoglycoside resistance genes

The frequency of the studied AME genes among *Enterococcus* species is shown in Table 1. Of the 17 *E. faecium* isolates with high-level gentamicin resistance (HLGR), *aac(6′)*-*Ie*-*aph(2″)*-*Ia* was the most prevalent (n = 12, 70.6%), followed by *aph(3′)*-*IIIa* (n = 9, 52.9%) and *aph(2″)*-*Id* (n = 4, 23.5%). However, the frequencies of resistance genes among the 20 HLGR *E. faecalis* isolates were as follows: *aph(3′)*-*IIIa* (n = 15, 75.0%), *aac(6′)*-*Ie*-*aph(2″)*-*Ia* (n = 12, 60.0%) and *aph(2″)*-*Id* (n = 3, 15.0%). Neither the *aph(2″)*-*Ib* nor the *aph(2″)*-*Ic* gene was detected among all of the HLGR isolates. No significant differences in the distribution of the evaluated AME genes were observed between *E. faecalis* and *E. faecium* (see Additional file 3: Table S3). Additionally, 31 HLGR enterococcal isolates (83.8%) carried two or more different AME genes (see Table 1). Moreover, 3 HLGR isolates (8.1%) and all of the gentamicin-susceptible isolates were negative for the examined AME genes.

**Table 1:** Distribution of aminoglycoside modifying enzyme genes in enterococci

AME gene	Distribution of HLGR in enterococci (n = 37)		Total no. (%) of isolates
	*E. faecalis* (n = 20)	*E. faecium* (n = 17)	
*aac(6′)*-*Ie*-*aph(2″)*-*Ia*	12	12	24 (64.9%)
*aph(2″)*-*Ib*	–	–	–
*aph(2″)*-*Ic*	–	–	–
*aph(2″)*-*Id*	3	4	7 (18.9%)
*aph(3′)IIIa*	15	9	24 (64.9%)
*aac(6′)*-*Ie*-*aph(2″)*-*Ia *+ *aph(2″)*-*Id*	2	3	5 (13.5%)
*aac(6′)*-*Ie*-*aph(2″)*-*Ia *+ *aph(3′)IIIa*	8	6	14 (37.8%)
*aph(2″)*-*Id *+ *aph(3′)IIIa*	3	4	7(18.9%)
*aac(6′)*-*Ie*-*aph(2″)*-*Ia *+ *aph(2″)*-*Id *+ *aph(3′)IIIa*	2	3	5(13.5%)

#### Distribution of erythromycin resistance genes

Table 2 shows the presence of erythromycin resistance genes among erythromycin- resistant enterococcus isolates. The frequencies of the erythromycin resistance genes were as follows: *erm(B)* (n = 44, 67.7%)*, erm(A)* (n = 3, 4.6%), and *erm(C)* (n = 1, 1.5%). Of the 31 erythromycin-resistant isolates of *E. faecium*, the majority of the isolates were positive for *erm(B)* (n = 17, 54.8%), followed by *erm(A)* (n = 3, 9.7%) and *erm(C)* (n = 1, 3.2%). Similarly, of the 33 erythromycin-resistant isolates of *E. faecalis*, 26 isolates (78.8%) carried *erm(B)*, while neither *erm(A)* nor *erm(C)* was detected. No significant differences in the distribution of the erythromycin resistance genes were identified between *E. faecalis* and *E. faecium* (see Additional file 3: Table S3). In 1 isolate (100%) of erythromycin-resistant *E. raffinosus,* the *erm(B)* gene was detected. Furthermore, only 1 isolate (1.5%) of the erythromycin-resistant enterococci contained both *erm(B)* and *erm(A)*. However, 17 isolates (26.2%) did not carry any of the examined *erm* genes. Our results revealed that the isolates of erythromycin-susceptible enterococci were negative for the *erm* genes examined in this study.

**Table 2:** Distribution ofErythromycin resistance genes in enterococci

Gene	Distribution of Erythromycin resistance in enterococci (n = 65)			Total no.(%) of isolates
	*E. faecalis* (n = 33)	*E. faecium* (n = 31)	*E. raffinosus* (n = 1)	
*erm(A)*	–	3	–	3 (4.6%)
*erm(B)*	26	17	1	44(67.7%)
*erm(C)*	–	1	–	1 (1.5%)
*erm(A) *+ *erm(B)*	–	1	–	1 (1.5%)

#### Distribution of the tetracycline resistance gene

Table 3 shows the frequency of the *tetM* gene among the 36 tetracycline- resistant enterococcal isolates, including 14 isolates of *E. faecium* and 22 isolates of *E. faecalis*. All of these tetracycline-resistant strains of enterococci were found to be positive for the *tetM* gene. The *tetM* gene was absent in all the tetracycline-susceptible isolates.

**Table 3:** Distribution of tetracycline resistance genes in enterococci

gene	Distribution of tetracycline resistance in enterococci (n = 36)		Total no.(%) of isolates
	*E. faecalis* (n = 22)	*E. faecium* (n = 14)	
*tetM*	22	14	36 (100%)

### Discussion

It has been shown that enterococci are opportunistic nosocomial pathogens capable of causing various infectious diseases. Many *Enterococcus* species have been reported, and *E. faecium and E. faecalis* are the most common human infectious strains of Enterococcus [14, 15]. This finding is similar to that observed in the present report.

It appears that the increased prevalence of multi-drug-resistant *Enterococcus* species has become a major public health problem. In the current study, a high percentage of enterococci exhibited resistance to erythromycin (89.0%), ciprofloxacin (74.0%), ampicillin (54.8%), gentamicin (high-level) (50.7%), tetracycline (49.3%) and nitrofurantoin (24.7%). However, the isolates were highly sensitive to the following antibiotics: teicoplanin (0 resistant strains), linezolid (0 resistant strains), and vancomycin (0 resistant strains), similar to other reports [16]. In this study, the rate of ampicillin resistance in *E.* *faecium* species showed a significant difference when compared to that of *E.* *faecalis*, which is in agreement with previous studies [17].

The presence of AME genes, such as *aac(6′)*-*Ie*-*aph(2″)*-*Ia, aph(2″)*-*Ib, aph(2″)*-*Ic, aph(2″)*-*Id,* and *aph(3′)IIIa*, that are responsible for high-level gentamicin resistance, have been extensively reported [18]. Our previous studies found that *aac(6′)*-*Ie*-*aph(2″)*-*Ia* was the most common AME gene [15]. In the current study, a high prevalence of the *aph(3′)*-*IIIa* gene was also found. We further showed that 37.8% of the HLGR enterococcal isolates carried both *aac(6′)*-*Ie*-*aph(2″)*-*Ia* and *aph(3′)*-*IIIa*, which was higher than our previous results [15]. Moreover, 8.1% of the HLGR enterococcal isolates did not carry any of the examined AME genes. This was lower than that previously reported by Li et al. [19].

The presence of a wide range of erythromycin resistance genes in *Enterococcus* species has been reported elsewhere [20]. In the present study, the most abundant *erm* gene was *erm(B)*, followed by *erm(A)* and *erm(C)*. A Previous study by Quiñones Pérez et al. found that 70.9% of erythromycin-resistant enterococcal isolates examined in their study carried *erm(B)* [21]. This value is close to that obtained in our present study (67.7%). In addition, the *erm(A)* and *erm(C)* genes were only detected in erythromycin-resistant isolates of *E. faecium*, which was a rarer occurrence than that previously reported [22]. Moreover, 17 isolates (26.2%) of the erythromycin- resistantent erococci were negative for the *erm* genes examined in this study. It is possible that other genes could be associated with erythromycin- resistant enterococcal isolates, such as *erm(D)*, *erm(E)*, *erm(F)*, *erm(G)*, *erm(Q)*, and the macrolide efflux pump (*msrA*).

Acquired resistance to tetracyclines in enterococci is often by mobile genetic elements [23]. The detection of the *tetM* gene by PCR has frequently been used to monitor tetracycline resistance in microbial populations [24]. The results of this study showed that 100% of the tetracycline-resistant *Enterococcus* isolates carried the *tetM* gene, which was higher than previously reported prevalences [25].

### Conclusions

*Enterococcus* species have become a significant cause of hospital-acquired infections. The present study showed that enterococci recovered from clinical samples in Baotou, China, contained a variety of antimicrobial resistance genes. These results will be helpful in clarifying the transmission mechanisms of antibiotic-resistant Enterococcus species.

## Limitations

This study tried to address the distribution of acquired antibiotic resistance genes among *Enterococcus* species isolated from a hospital in Baotou, China. However, the study was not without limitations. This was a small study that could not include additional samples of *Enterococcus* species taken from this region. In addition, the study utilized PCR-based methods to detect some common acquired antibiotic resistance genes, and tests for several additional antibiotic resistance genes will be needed.

## Additional files

**Additional file 1: Table S1.** PCR primers used in the amplification of resistance genes.

**Additional file 2: Table S2.** The resistance rate of the clinical isolates of enterococci species to various antimicrobial agents.

**Additional file 3: Table S3.** Differences in the prevalence of resistance genes between *E. faecalis* and *E. faecium* were compared using the Chi square test, with a *p* value < 0.05 indicating statistical significance.

## Abbreviations

K–B assay: Kirby–Bauer assay
AME: aminoglycoside-modifying enzyme
CLSI: Clinical and Laboratory Standards Institute
HLGR: high-level gentamicin resistance
