# Supplementary material for: Distribution of acquired antibiotic resistance genes among Enterococcus spp. isolated from a hospital in Baotou, China
Source: BMC Res Notes. 2019 Jan 15;12:27. doi: 10.1186/s13104-019-4064-z (PMC6334421; doi:10.1186/s13104-019-4064-z)
Supplement: Supplementary file 1 — Additional file 1: Table S1. PCR primers used in the amplification of resistance genes. [file 13104_2019_4064_MOESM1_ESM.docx]

**Distribution of Acquired Antibiotic Resistance Genes Among *Enterococcus* spp. Isolated from a Hospital in Baotou, China**

Yingjie Tian, Hui Yu, and Zhanli Wang*

The Second Affiliated Hospital, Baotou Medical College, 30 Hude Mulin Street, Baotou 014030, China

**Additional file 1:**

Table S1. PCR primers used in the amplification of resistance genes.

| Gene | Primers（5ˊ-3ˊ） | Size  (bp) | Annealing temperature  (◦C ) | Reference |
| --- | --- | --- | --- | --- |
| *aac(6')-Ie-aph(2'')-Ia* | F：CAGGAATTTATCGAAAATGGTAGAAAAG  R：CACAATCGACTAAAGAGTACCAATC | 369 | 58 | [10] |
| *aph(2'')-Ib* | F：CTTGGACGCTGAGATATATGAGCAC  R：GTTTGTAGCAATTCAGAAACACCCTT | 867 | 56 | [10] |
| *aph(2'')-Ic* | F：CCACAATGATAATGACTCAGTTCCC  R：CCACAGCTTCCGATAGCAAGAG | 444 | 56 | [10] |
| *aph(2'')-Id* | F：GTGGTTTTTACAGGAATGCCATC  R：CCCTCTTCATACCAATCCATATAACC | 641 | 56 | [10] |
| *aph(3')-IIIa* | F：GGCTAAAATGAGAATATCACCGG  R：CTTTAAAAAATCATACAGCTCGCG | 523 | 58 | [10] |
| *erm(A)* | F：GTTCAAGAACAATCAATACAGAG  R：GGATCAGGAAAAGGACATTTTA | 421 | 56 | [11] |
| *erm(B)* | F：CATTTAACGACGAAACTGGC  R：GGAACATCTGTGGTATGGCG | 405 | 58 | [12] |
| *erm(C)* | F：GCTAATATTGTTTAAATCGTCAATTCC  R：GGATCAGGAAAAGGACATTTTAC | 572 | 58 | [11] |
| *tetM* | F：GTGTGACGAACTTTACCGAA  R：GCTTTGTATCTCCAAGAACAC | 501 | 58 | [13] |
