# Supplementary material for: Distribution of acquired antibiotic resistance genes among Enterococcus spp. isolated from a hospital in Baotou, China
Source: BMC Res Notes. 2019 Jan 15;12:27. doi: 10.1186/s13104-019-4064-z (PMC6334421; doi:10.1186/s13104-019-4064-z)
Supplement: Supplementary file 2 — Additional file 2: Table S2. The resistance rate of the clinical isolates of enterococci species to various antimicrobial agents. [file 13104_2019_4064_MOESM2_ESM.docx]

**Distribution of Acquired Antibiotic Resistance Genes Among *Enterococcus* spp. Isolated from a Hospital in Baotou, China**

Yingjie Tian, Hui Yu, and Zhanli Wang*

The Second Affiliated Hospital, Baotou Medical College, 30 Hude Mulin Street, Baotou 014030, China

**Additional file 2:**

Table S2. The resistance rate of the clinical isolates of enterococci species to various antimicrobial agents.

| Antibiotic | *E. faecalis*  (n=35) | | | *E. faecium*  (n=36) | | | *E. gallinarum*  (n=1) | | | | *E. raffinosus*  (n=1) | | | | Total no.(%) of isolates  (n=73) | | | |
| --- | --- | --- | --- | --- | --- | --- | --- | --- | --- | --- | --- | --- | --- | --- | --- | --- | --- | --- |
|  | R* | I* | S* | R | I | S | R | I | S | R | | I | S | R | | I | S |  |
| Gentamicin（High-level） | 20 | - | 15 | 17 | - | 19 | - | - | - | - | | - | - | 37(50.7%) | | - | 34(46.6%) |  |
| Ampicillin | 14 | - | 21 | 26 | - | 10 | - | - | - | 1 | | - | - | 40(54.8%) | | - | 31(42.5%) |  |
| Tecolanin | - | - | - | - | - | - | - | - | - | - | | - | - | - | | - | - |  |
| Vancomycin | - | - | - | - | - | - | - | - | - | - | | - | - | - | | - | - |  |
| Erythromycin | 33 | 1 | 1 | 31 | 2 | 3 | - | 1 | - | 1 | | - | - | 65(89.0%) | | 4(5.5%) | 4(5.5%) |  |
| Linezolid | - | - | - | - | - | - | - | - | - | - | | - | - | - | | - | - |  |
| Ciprofloxacin | 29 | 3 | 3 | 24 | 1 | 11 | 1 | - | - | - | | - | - | 54(74.0%) | | 4(5.5%) | 14(19.2%) |  |
| Tetracycline | 22 | - | 13 | 14 | 1 | 21 | - | - | - | - | | - | - | 36(49.3%) | | 1(1.4%) | 34(46.6%) |  |
| Nitrofurantoin | 7 | 1 | 27 | 11 | 5 | 20 | - | - | - | - | | - | - | 18(24.7%) | | 6(8.2%) | 47(64.4%) |  |

Notes: ***** R (resistance); I (intermediate); S (susceptible).
