# Supplementary material for: Distribution of acquired antibiotic resistance genes among Enterococcus spp. isolated from a hospital in Baotou, China
Source: BMC Res Notes. 2019 Jan 15;12:27. doi: 10.1186/s13104-019-4064-z (PMC6334421; doi:10.1186/s13104-019-4064-z)
Supplement: Supplementary file 3 — Additional file 3: Table S3. Differences in the prevalence of resistance genes between E. faecalis and E. faecium were compared using the Chi square test, with a p value < 0.05 indicating statistical significance. [file 13104_2019_4064_MOESM3_ESM.docx]

**Distribution of Acquired Antibiotic Resistance Genes Among *Enterococcus* spp. Isolated from a Hospital in Baotou, China**

Yingjie Tian, Hui Yu, and Zhanli Wang*

The Second Affiliated Hospital, Baotou Medical College, 30 Hude Mulin Street, Baotou 014030, China

**Additional file 3:**

Table S3. Differences in the prevalence of resistance genes between *E. faecalis* and *E. faecium* were compared using the Chi-square test, with a p-value < 0.05 indicating statistical significance.

| Gene | *E. faecalis* | *E. faecium* | *χ^2^* | *P* |
| --- | --- | --- | --- | --- |
| *aac(6')-Ie-aph(2'')-Ia* | 12 | 12 | 0.452 | 0.731 |
| *aph(2'')-Ib* | - | - | - | - |
| *aph(2'')-Ic* | - | - | - | - |
| *aph(2'')-Id* | 3 | 4 | 0.057 | 0.811 |
| *aph(3')IIIa* | 15 | 9 | 1.962 | 0.188 |
| *aac(6')-Ie-aph(2'')-Ia+aph(2'')-Id* | 2 | 3 | 0.038 | 0.644 |
| *aac(6')-Ie-aph(2'')-Ia+aph(3')IIIa* | 8 | 6 | 0.087 | 1.000 |
| *aph(2'')-Id+aph(3')IIIa* | 3 | 4 | 0.057 | 0.811 |
| *aac(6')-Ie-aph(2'')-Ia+aph(2'')-Id+aph(3')IIIa* | 2 | 3 | 0.038 | 0.644 |
| *tetM* | 22 | 14 | - | - |
| *erm(A)* | 0 | 3 | 1.535 | 0.215 |
| *erm(B)* | 26 | 17 | 4.159 | 0.062 |
| *erm(C)* | 0 | 1 | 0.001 | 0.975 |
| *erm(A)+erm(B)* | 0 | 1 | 0.001 | 0.975 |
